# Antibiotic Prescriptions for Respiratory Tract Viral Infections in the Colombian Population

**DOI:** 10.3390/antibiotics10070864

**Published:** 2021-07-16

**Authors:** Manuel E. Machado-Duque, Diego Arturo García, Melissa Hiromi Emura-Velez, Andrés Gaviria-Mendoza, Claudia Giraldo-Giraldo, Jorge E. Machado-Alba

**Affiliations:** 1 Grupo de Investigación en Farmacoepidemiología y Farmacovigilancia, Universidad Tecnológica de Pereira-Audifarma S.A., Pereira 660003, Colombia; memachado@utp.edu.co (M.E.M.-D.); angaviria@utp.edu.co (A.G.-M.); claudiag@audifarma.com.co (C.G.-G.); 2 Grupo de Investigación Biomedicina, Fundación Universitaria Autónoma de las Américas, Pereira 660003, Colombia; dagospina.50@gmail.com (D.A.G.); melissa_emura@hotmail.com (M.H.E.-V.)

**Keywords:** respiratory tract infections, antibiotics, primary care, pharmacoepidemiology

## Abstract

Antimicrobials are frequently inappropriately prescribed for the management of upper respiratory tract infections (URTIs); therefore, the frequency of antibiotic prescriptions for patients with viral URTIs was assessed in this study. A cross-sectional study, including ambulatory patients diagnosed with viral URTI, was conducted, and records of antimicrobial prescriptions were obtained. Sociodemographic, clinical (diagnostic), and pharmacological (antimicrobial) variables were assessed. Through multivariate analysis, variables associated with the use of antibiotics for viral infections were identified. A total of 341,182 patients with viral URTIs were identified. The patients, who were from 26 different departments of Colombia, had a mean age of 29.7 ± 23.5 years and a female predominance of 58.7% (*n* = 200,195). The most frequent viral infections were as follows: acute rhinopharyngitis (common cold) (*n* = 206,211; 60.4%); unspecified acute tonsillitis (*n* = 27,432; 8.0%); and acute pharyngitis (*n* = 26,411; 7.7%). A total of 24.8% of the patients (*n* = 84,453) received a prescription for antibiotics, predominantly penicillins (*n* = 61,871; 18.1%) and cephalosporins (*n* = 10,926; 3.2%). Patients treated in Atlántico, Valle, and Risaralda departments, along with those older than 5 years, were more likely to receive antibiotics for the treatment of viral infections. Antibiotics are frequently prescribed for the management of URTIs, which is considered an inappropriate practice due to a lack of clinical benefits, increased generation of antimicrobial resistance, and a risk of adverse reactions due to the use of medications that patients do not require. Drug utilization studies are a great tool for monitoring how antibiotics are being used and planning interventions to improve their use.

## 1. Introduction

Currently, respiratory tract infections necessitate numerous consultations throughout different services of health institutions; in the United States, approximately 43 million annual outpatient visits are related to symptoms, such as cough and odynophagia [1]. These diseases are caused by pathogens, such as bacteria, fungi (immunosuppressed host), and viruses. Viruses are the main causes, as evidenced in the systematic analysis conducted in the 2016 Global Burden of Disease Study, where lower respiratory tract infections generated 32.2 deaths for every 100,000 people [2,3]. The most frequent upper respiratory infections are rhinopharyngitis (39.7%), pharyngitis (14.4%), and bronchitis (12.6%) [4] caused by rhinovirus, coronavirus, adenovirus, influenza virus, respiratory syncytial virus (RSV), and enterovirus, among others [5].

The current approach to the clinical management of respiratory tract infections is based on symptom control, and in most conditions, the use of antibiotics shows no benefit [6,7,8]. This is reflected in a study that was conducted in 12 European countries, where no evidence of significant symptomatic improvement was found in a comparison of amoxicillin with placebo for the treatment of viral infections; only significant differences in the presence of bacterial coinfection were identified (hazard ratio (HR): 0.24 [0.11–0.53] *p* < 0.001), as expected [9].

Therefore, the use of antimicrobials in patients with viral infections may be inappropriate. Donnelly et al. described the frequency of antibiotic use in the United States and found antimicrobial prescriptions for 61.1% of patients with diagnoses of upper respiratory tract infections, 47.9% of which were inappropriate [10]. A study by Ebell et al. performed in the United States found that 49.4% of patients with upper respiratory infections were prescribed antibiotics, with this practice being more common in those diagnosed with acute bronchitis (67.8%). In addition, this study found a higher rate of prescription of these drugs for patients older than 65 years and a slightly greater antibiotic prescription rate for viral infections in an emergency setting (49.4%) than in a primary care setting (47.9%) [11].

Inappropriate antibiotic use is associated with increased adverse events, respiratory tract infection complications, repeated visits, and increased bacterial resistance [12]. Furthermore, this practice increases costs for the health system in general, as is the case in the United States, where the estimated cost overruns are approximately USD 4.6 billion/year [13], and in the European health system, where the estimated extra cost is EUR 1.5 billion due to the induction of bacterial resistance [14].

The Colombian health system offers universal coverage to the entire population and has a benefits plan that includes many of the antimicrobials commonly used in clinical practice without great limitations on their prescription. Therefore, the objective of this study was to determine the frequency of antibiotic prescriptions for patients with viral infections during 2018.

## 2. Results

A total of 341,182 patients distributed throughout 26 departments in Colombia and diagnosed with viral infection were identified. The mean age of the patients was 29.7 ± 23.5 years, and 58.7% (*n* = 200,195) were women. Table 1 shows the distribution by age group and department.

The most frequent viral infections were acute rhinopharyngitis (common cold), unspecified acute tonsillitis, and acute pharyngitis. A total of 24.8% of all patients diagnosed with a viral infection received a prescription for an antimicrobial, particularly penicillins, cephalosporins, and macrolides (see Table 2).

**Figure 1:**
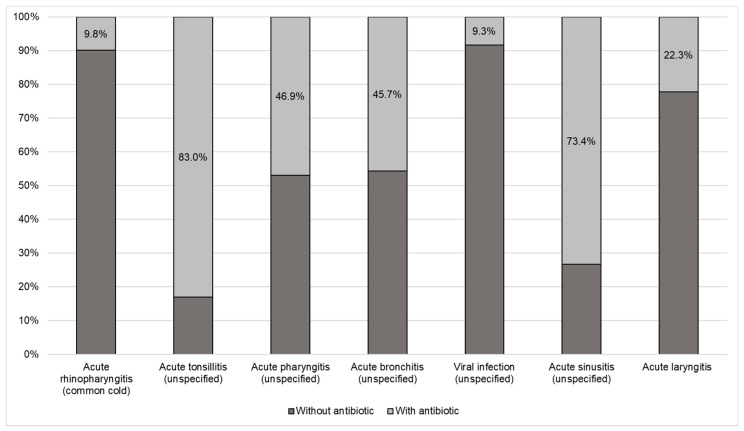
Proportion of antibiotic use according to the most frequent diagnoses in patients with viral respiratory tract infections, Colombia—2018.

**Figure 2:**
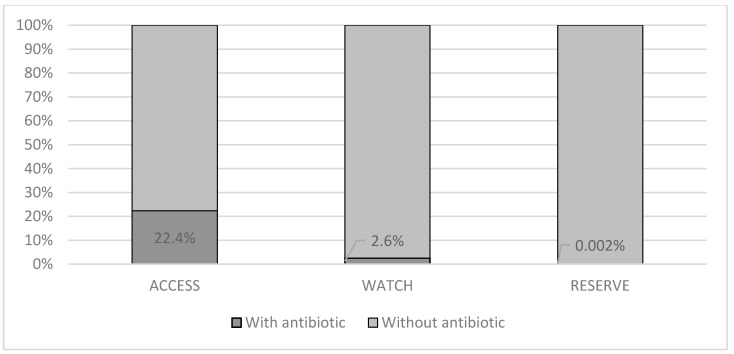
Distribution of use of different antibiotics according to the AWaRe classification (WHO 2019) by patients with viral respiratory tract infections, Colombia—2018.

Figure 1 shows the proportion of antibiotic use for each of the diagnoses, where antibiotics were most frequently prescribed for tonsillitis, sinusitis, pharyngitis, and acute bronchitis and less frequently for the common cold. Figure 2 shows the proportion of antimicrobial use according to the AWaRe classification (WHO 2019) for patients with an infection of viral origin; notably, in most cases, the antibiotics were classified in the access group, which are the antibiotics of choice for the 25 most common infections and those that are more widely available with less concern for antimicrobial resistance.

### Multivariate Analysis

Exploratory logistic regression adjusted for age and sex indicated that patients who were treated in departments in Atlantico, Valle del Cauca, and Risaralda and all age groups older than 5 years were more likely to receive antibiotics to treat viral infections, while those treated in departments in Antioquia and Bolivar had a lower risk of receiving inappropriate antibiotic prescriptions (Hosmer–Lemeshow test *p* = 0.000, Nagelkerke’s R squared = 0.025) (Table 3).

## 3. Discussion

This study enabled the estimation of the frequency of the use of nonindicated antimicrobials in a quarter of patients diagnosed with viral infections, with inappropriate prescriptions being more common for those diagnosed with tonsillitis and acute sinusitis. These data have not yet been described for the Colombian population and can be useful for the development of continuing education strategies and decision making regarding appropriate antibiotic use.

Compared to children younger than 5 years, all patients older than 5 years had a higher probability of receiving antibiotics for the management of respiratory infections of viral origin, which has already been described by other authors as an inappropriate prescription practice [15,16]. The reasons for this situation were not established; however, the higher frequency of prescribing antibiotics in some regions of Colombia may be related to differences in diagnostic and medical resources, access to specialist care, pressure from patients or patients’ families who request antibiotics, and physicians’ concern about adequately treating a possible bacterial infection [17]. In addition, the expectations of parents of children with these infections have been reported to be a factor contributing to inappropriate antibiotic use [18]. Therefore, new studies must be developed to determine the reasons and possible explanations for the nonindicated use of antimicrobials for viral infections.

In the United States in 2016, Chua et al. found a proportion of inappropriate antibiotic use by patients with respiratory infections similar to that obtained in this study (23.9%) [15], in a study by Fleming-Dutra et al. in 2011 in the United States (30% nonindicated use) [16], and in a study by Ray M et al. who reported 25.0% inappropriate use in 2015 in the same country [19]. These findings demonstrate the need for strategies to improve diagnostic processes for conditions in which differentiating a viral infection from a bacterial infection is difficult, such as viral pneumonia or pharyngitis. In addition, with more advanced diagnostic techniques, better and appropriate use of antimicrobials can be promoted [20,21]. Inappropriate use of antibiotics for certain respiratory infections, such as acute sinusitis or pharyngitis, can be explained by poor classification at the time of diagnosis, which can influence decision making by the doctor, who believes that an antimicrobial is required [16,20,21], which not only is unnecessary but also increases the known risks of generating resistance and worse clinical outcomes [22].

The most frequently used antibiotics were beta-lactams, which are considered quite safe for patients but are also regularly used for many types of bacterial infections, ranging from those located in the urinary tract to pneumonias and meningitis, which raises concern about the risk of generating resistance that may be associated with their nonindicated use for viral infections. This can be considered a public health problem, as described above [23]. Other studies have also shown that penicillins and cephalosporins are frequently prescribed inappropriately [15,16,23]. Importantly, drugs for exclusive use in severe or critical conditions, such as carbapenems, were prescribed (*n* = 6), especially for rhinopharyngitis and acute pharyngitis (*n* = 4). The same was observed with fluoroquinolones, which are associated with adverse reactions, some of which are severe. This leads regulatory agencies and the World Health Organization to limit their use for infections for which the doctor is certain of the benefit–risk ratio; therefore, their use is not justified for infections of viral origin [24,25,26,27]. Another group identified in this analysis was aminoglycosides, which are recommended as second-line drugs for Gram-negative infections. These drugs are associated with safety problems and adverse reactions, such as kidney damage and injury to the eighth cranial nerve, among others [15,22,28].

The differences found for the inappropriate use of antibiotics for viral infections between different departments throughout the country are common findings in pharmacoepidemiological studies. These findings can be explained by differences in physician training, prescription habits, regional management guidelines, and even in the frequency of presentation of different infections, which has already been described in this type of research [27,29]. In regions of the country where more frequent inappropriate use was found, continuing education interventions that improve prescription habits should be prioritized to achieve better health outcomes and lower rates of antimicrobial resistance.

Some limitations of the design of this type of research are recognized, such as the lack of a review of the medical records of each patient to identify clinical conditions.. This includes symptom duration, the severity of infection, and the number of visits to the doctor, along with the lack of paraclinical analyses with the results of cultures, leukograms, and even the state of immunocompetence that may have led clinicians to make antibiotic prescription decisions. In addition, the findings can be extrapolated only to populations with similar insurance characteristics.

## 4. Materials and Methods

This was a cross-sectional study of patients of any age and sex diagnosed with infection of viral origin recorded using the codes of the International Classification of Diseases version 10.0 (ICD-10), including otitis media (OM), bronchitis, bronchiolitis, sinusitis, pharyngitis, tonsillitis, laryngitis, tracheitis, nasopharyngitis, and mastoiditis (see ICD-10 codes in annex 1). The patients were covered by one of four EPSs (Entidad Promotora de Salud, the Colombian equivalent to a health insurance company) that cover approximately 6.5 million people for outpatient consultations (primary care) or priority care (priority outpatient consultation) in different departments of Colombia between 1 January and 31 December 2018. This observation period was selected due to data availability at the time of the analysis and study design. For each patient with a viral infection identified, dispensing records were searched using the same consultation period for prescriptions for antibiotics (Anatomical, Therapeutic, Chemical Classification System (ATC) codes) (penicillins, cephalosporins, macrolides, fluoroquinolones, tetracyclines, sulfonamides, and lincosamides). No exclusion criteria were considered.

All information was obtained from the medication dispensing records of Audifarma S.A. (Drug-claim database. Audifarma S.A is currently the largest drug dispensing company in the Colombian health system). This database also recorded the primary and secondary diagnoses (ICD-10) related to the prescription for each drug dispensed. The classification of diagnoses was made by the prescriber during the physician office visits. A database including the following variables was constructed:

1. Sociodemographic: age, sex, city and department of residence, health insurer, and care service;

2. Clinical: viral pathologies diagnosed and recorded using ICD-10 codes;

3. Pharmacological: antibiotics prescribed according to ATC codes and categorized by group as follows: (a) tetracyclines (J01A); (b) beta-lactams and other beta-lactams and penicillins (J01C–J01D) (including penicillins, cephalosporins, carbapenems, and aztreonam); (c) sulfonamides and trimethoprim (J01E); (d) macrolides, lincosamides, and streptogramins (J01F); (e) aminoglycosides (J01G); (f) fluoroquinolones (J01M); and (g) other antibacterials and associated drugs (J01R and JO1X). The antibiotics used in the patients were categorized according to the WHO AWaRe classification and each antimicrobial in access, watch, and reserve.

The protocol was approved by the Bioethics Committee of Universidad Tecnológica de Pereira in the category of “research without risk”. Based on resolution No. 8430 of 1993 of the Ministry of Health of Colombia, the principles established by the Declaration of Helsinki were respected. In no case were personal data of the patients used.

### Analysis

The database was developed in Microsoft Excel 2016 for Windows, and the analyses were carried out using the statistical package SPSS 25.0 (IBM—New York, NY, USA), with descriptive statistics (frequencies and proportions) for categorical variables and measures of central tendency and dispersion for continuous variables. In addition, bivariate analyses were performed, applying the *X*^2^ test for categorical variables. An exploratory binary logistic regression model was applied considering the prescription of antibiotics for viral infection (yes/no) as the dependent variable. The enter method was used to include the covariates, including variables that were statistically associated in the bivariate analysis and variables considered potential confounders (age, sex, and theoretical/biological plausibility that could explain the results). Covariates were tested for interactions and multicollinearity. The Hosmer–Lemeshow test and Nagelkerke’s R squared were calculated in order to describe the goodness of fit. Statistical significance was established as *p* < 0.05.

## 5. Conclusions

The above findings indicate that a quarter of patients covered by four insurers in Colombia who sought medical consultations for viral infections received antibiotics. This practice should be considered inappropriate because of the lack of clinical benefits and an increase in the risk of resistance by microorganisms, in addition to the risk of adverse reactions associated with the use of medications that patients do not require. These results provide a basis for new studies investigating the reasons why doctors use antibiotics without a sufficient foundation to generate educational strategies that improve the use of this group of drugs.

## Figures and Tables

**Table 1:** Sociodemographic characteristics and regions of origin of patients with viral respiratory tract infections, Colombia—2018.

Variable	Frequency	%
Age (mean ± SD)	29.7 ± 23.5	
Female	200,195	58.7
Age group		
Infants < 5 years old	68,496	20.1
Children and adolescents 5–18 years old	63,837	18.7
Young adults 18–45 years old	111,748	32.8
Older adults 45 to 65 years old	65,536	19.2
Elderly individuals over 65 years old	31,565	9.3
**Region**		
Bogota	164,157	48.1
Atlantico	31,644	9.3
Valle del Cauca	20,814	6.1
Bolivar	20,621	6.0
Antioquia	16,351	4.8
Risaralda	13,155	3.9
Magdalena	12,372	3.6
Cundinamarca	11,905	3.5
Caldas	10,073	3.0
Other regions	40,090	11.8

**Table 2:** Most frequent diagnoses and groups of antibiotics prescribed for patients with viral respiratory tract infections, Colombia—2018.

Variable	Frequency *n* = 341,182	%
Use of antibiotics in viral infections (patients)	84,453	24.8
**Groups of antibiotics used**		
Penicillins	61,871	18.1
Cephalosporins	10,926	3.2
Macrolides	6400	1.9
Sulfonamides	2302	0.7
Fluoroquinolones	2201	0.6
Tetracyclines	1477	0.4
Aminoglycosides	279	0.1
Lincosamides	59	0.01
Carbapenems	6	0.001
**Viral infection diagnoses**		
Acute rhinopharyngitis (common cold)	206,211	60.4
Acute tonsillitis (unspecified)	27,432	8.0
Acute pharyngitis (unspecified)	26,411	7.7
Acute bronchitis (unspecified)	21,424	6.3
Viral infection (unspecified)	15,578	4.6
Acute sinusitis (unspecified)	9897	2.9
Acute laryngitis	8632	2.5
Other diagnoses (89 total types)	25,597	7.5

**Table 3:** Multivariate logistic regression analysis identifying variables associated with the use of antibiotics in a group of patients with viral respiratory tract infections, Colombia—2018.

			Confidence Interval (95%)	
Variable	Statistical Significance	OR	Lower	Upper
Female sex	0.350	0.992	0.976	1.009
Infants < 5 years	Ref.	Ref.		
Children and adolescents aged 5–18 years	<0.001	1.666	1.622	1.712
Young adults aged 18–45 years	<0.001	2.022	1.973	2.072
Older adults aged 45–65 years	<0.001	1.849	1.799	1.900
Elderly older than 65 years	<0.001	1.633	1.580	1.689
Treated in Bogota	0.279	1.011	0.991	1.032
Treated in Atlantico	0.020	1.037	1.006	1.070
Treated in Valle del Cauca	<0.001	1.152	1.112	1.193
Treated in Bolivar	<0.001	0.798	0.768	0.829
Treated in Antioquia	<0.001	0.577	0.552	0.604
Treated in Risaralda	<0.001	1.783	1.714	1.855

OR: Odds ratio.

## Data Availability

protocolos.oi dx.doi.org/10.17504/protocols.io.bwjmpck6.
